# Automated Pulmonary Embolism Risk Assessment Using the Wells Criteria: Validation Study

**DOI:** 10.2196/32230

**Published:** 2022-02-28

**Authors:** Nasen Jonathan Zhang, Philippe Rameau, Marsophia Julemis, Yan Liu, Jeffrey Solomon, Sundas Khan, Thomas McGinn, Safiya Richardson

**Affiliations:** 1 Northwell Health Manhasset, NY United States; 2 Latin American School of Medicine Havana Cuba

**Keywords:** health informatics, pulmonary embolism, electronic health record, quality improvement, clinical decision support systems

## Abstract

**Background:**

Computed tomography pulmonary angiography (CTPA) is frequently used in the emergency department (ED) for the diagnosis of pulmonary embolism (PE), while posing risk for contrast-induced nephropathy and radiation-induced malignancy.

**Objective:**

We aimed to create an automated process to calculate the Wells score for pulmonary embolism for patients in the ED, which could potentially reduce unnecessary CTPA testing.

**Methods:**

We designed an automated process using electronic health records data elements, including using a combinatorial keyword search method to query free-text fields, and calculated automated Wells scores for a sample of all adult ED encounters that resulted in a CTPA study for PE at 2 tertiary care hospitals in New York, over a 2-month period. To validate the automated process, the scores were compared to those derived from a 2-clinician chart review.

**Results:**

A total of 202 ED encounters resulted in a completed CTPA to form the retrospective study cohort. Patients classified as “PE likely” by the automated process (126/202, 62%) had a PE prevalence of 15.9%, whereas those classified as “PE unlikely” (76/202, 38%; Wells score >4) had a PE prevalence of 7.9%. With respect to classification of the patient as “PE likely,” the automated process achieved an accuracy of 92.1% when compared with the chart review, with sensitivity, specificity, positive predictive value, and negative predictive value of 93%, 90.5%, 94.4%, and 88.2%, respectively.

**Conclusions:**

This was a successful development and validation of an automated process using electronic health records data elements, including free-text fields, to classify risk for PE in ED visits.

## Introduction

Computed tomography pulmonary angiography (CTPA) is the gold standard test for diagnosing patients with pulmonary embolism (PE), a potentially deadly condition that often presents with nonspecific signs and symptoms [1,2]. Fast, sensitive, and specific, CTPA use has rapidly proliferated since it supplanted ventilation-perfusion scanning for the diagnosis of PE in the 1990s [3,4]. While CTPA testing is widely available and easy to perform, its utility must be weighed against harm from ionizing radiation and intravenous contrast. Potential harm from CTPA includes a 14% risk of contrast-induced nephropathy and lifetime radiation-induced malignancy risk as high as 2.76% [5,6].

Clinical prediction rules, such as the Wells criteria for pulmonary embolism, or the Wells score, have been developed and widely validated to assist providers with the decision to perform CTPA [7]. By estimating pretest probability and recommending CTPA only when suspicion is appropriately high, the use of such tools can reduce the number of tests performed without missing diagnoses of PE [8]. The incorporation of prediction rules into electronic health record (EHR) systems as clinical decision support (CDS) has been shown in multiple studies to significantly improve CTPA yield by 30% to 98% [9-11]. However, acceptance of CDS varies among physicians as CDS use is viewed as time-consuming [12]. At our institution, although users of a CDS tool incorporating the Wells score had CTPA yields of 38% higher than nonusers, the tool was dismissed in 65% of the cases [13]. Rather than requiring burdensome review of fragmented clinical data [14-16] and manual input of score components by providers, a CDS tool that presents a Wells score automatically calculated from existing EHR data could improve efficiency and usability and thereby tool acceptance [17,18].

Previous analysis of the Wells score concluded that it is less amenable to automatic calculation due to the inclusion of variables that either require clinical gestalt (PE as or more likely than alternative diagnosis) or are likely to be embedded in unstructured data (clinical signs and symptoms of deep venous thrombosis [DVT] and hemoptysis) [19]. Yet, using narrow definitions of the Wells score components based only on structured data can lead to decreased sensitivity for relevant clinical documentation [17]. The objective of our study was to design an automated process that incorporates information from unstructured data, and to validate its accuracy.

## Methods

### Study Setting and Design

This retrospective cohort study of emergency department (ED) encounters took place at 2 academic tertiary-care hospitals in New York. The study included all consecutive adult ED visits during the months of May and June of 2019 where a CTPA was performed to evaluate for PE. CTPA studies carried out for other indications, for example aortic dissection, were excluded. Patient demographic and clinical characteristics not part of the Wells Score were not collected. The study was approved by the institutional review board as minimal-risk research using data collected for routine clinical practice and the requirement for informed consent was waived. Data were collected from the enterprise EHR (Sunrise Clinical Manager, Allscripts, Chicago, IL, U.S.) reporting database.

### Automatic Score Design

We designed the automated process for the Wells score calculation with usability for an ED-based CDS tool as our goal. The process was therefore limited to only information in each encounter that was recorded prior to the CTPA order. We incorporated all 7 components of the Wells score, which are clinical signs and symptoms of DVT (3 points), PE being as or more likely than other diagnoses (clinical gestalt, 3 points), heart rate greater than 100 beats per minute (1.5 points), immobilization for at least 3 days or surgery in the prior 4 weeks (1.5 points), previous objectively diagnosed PE or DVT (1.5 points), hemoptysis (1 point), and active malignancy (with treatment within 6 months or palliative, 1 point).

Clinical signs and symptoms consistent with DVT or hemoptysis were taken from the chief complaint fields of the ED nurse triage note. This note is completed on patient arrival, before assessment by a provider, and includes both free text and discrete options for documentation of chief complaint. By combining a list of anatomic terms describing parts of the lower extremity, such as “leg” or “thigh,” terms of laterality, and prefix and suffix descriptors, such as “pain in” or “edema” (Textbox 1), we generated a list of 192 search phrases (Table S1 in Multimedia Appendix 1) for the signs and symptoms of DVT component of the Wells score. The list included common abbreviations referring to the lower extremity, such as “LE,” and included indicators of laterality. A similar list of 7 phrases describing hemoptysis (“hemoptysis,” “coughing blood,” “coughing up blood,” “blood-tinged sputum,” “bloody sputum,” “blood in sputum,” and “blood in phlegm”) was created. These lists were supplemented with phrasing encountered during a preliminary review of ED nurse triage notes and ED provider notes from encounters with CTPA in a period prior to our period of study.

**Terms used in combination to generate list of key phrases indicating signs and symptoms of deep venous thrombosis.**

List of anatomic terms describing parts of the lower extremity

- Prefix descriptors
  - edema to
  - swollen
  - pain in
- Indicators of laterality
  - bilateral
  - b/l
  - bl
  - right
  - left
  - L
  - R
- Anatomic terms
  - LE
  - lower extremity
  - leg
  - lower leg
  - thigh
  - calf
- Suffix descriptors
  - swelling
  - swollen
  - edema
  - pain
  - discomfort

We assumed 3 points for clinical gestalt for all encounters, assuming high concern for PE by the provider as a CTPA was performed. The remaining 4 components of the score were derived from structured data. For the heart rate criterion, the maximum value prior to the CTPA order was extracted, and 1.5 points were given if greater than 100. For the immobilization criterion, the EHR was queried for any intensive care unit stays and operative notes (specifying use of general anesthesia) within the preceding 30 days, as well as International Classification of Disease (ICD)-10 codes corresponding to quadriplegia. For history of PE/DVT, the problem list in Sunrise was queried for relevant ICD codes (for PE: ICD-9 codes 415.1, 415.11, 415.12, 415.13, 415.19, V12.55; ICD-10 code I26.99; and for DVT: ICD-9 codes 453.4-453.9, V12.51; ICD-10 code I82.409). For active malignancy, the Sunrise problem list was similarly queried for ICD-10 codes corresponding to a malignancy diagnostic group. The query was limited to problem list items documented prior to the index CTPA order. Once all score components became available, each encounter was classified as “PE likely” (Wells score greater than 4) or “PE unlikely” (Wells score less than or equal to 4) based on the two-tier model of risk stratification.

### Chart Review

A 2-reviewer manual chart review was conducted to validate the automatically derived Wells scores. A review process and standardized data collection sheet were first designed by the senior investigator and were trialed by 2 clinician-investigators (NZ and PR) in a preliminary review using data from a period prior to our period of study. In this preliminary review, the data collection process was refined and standardized. Subsequently, 2 investigators independently reviewed data from the study period. The review of each chart included the entirety of available data, including vital signs, laboratory values, radiology reports, problem list, and nurse and provider notes. Clinician notes were reviewed if they were linked to the patient encounter even if documentation was completed after CTPA order. It was assumed that the provider would have been aware of all findings documented in the history and physical exam before CTPA order. Three points were given for clinical gestalt in all cases. During the review, D-dimer ordering and the results of the CTPA (whether positive for PE) were also noted. Interreviewer agreement was measured by comparing risk classifications for encounters based on the Wells score using the Cohen kappa coefficient. Discrepancies were resolved by consensus.

### Measures and Data Analysis

The automated Wells score components based on the queries designed as above were then compared to manually derived score components as the gold standard to arrive at sensitivity and specificity data for each component, as well as sensitivity, specificity, positive predictive value, and negative predictive value with respect to risk classification based on the two-tier model. Positive and negative predictive values for the automatic score were also calculated with regards to risk stratification as “PE likely,” as this category is recommended to proceed directly to CTPA. For the lower risk category, a D-dimer is recommended to be performed first, and if normal, to stop further PE evaluation. To assess the ability of the automated score to stratify risk, we calculated the CTPA yield for each automated risk category. CTPA yield was calculated as the number of PE diagnoses divided by the number of CTPA exams. Ordering of the CTPA study was considered guideline concordant if the patient either had a Wells score in the “PE likely” category or in the “PE unlikely” category and if a D-dimer was subsequently ordered and was above the upper limit of normal (>230 ng/mL). Otherwise, the CTPA order was considered not guideline concordant. All data analysis was performed in Microsoft Excel.

## Results

### Prevalence of PE and Score Components

A total of 202 ED encounters resulted in a completed CTPA to form the retrospective study cohort. There was a high interreviewer agreement with a Cohen kappa 0.93. Based on chart review of CTPA results, the overall prevalence of PE was 12.9% (26/202 encounters). Patients classified as “PE unlikely” by the automated Wells score had a PE prevalence of 7.9% (6/76 encounters), whereas those classified as “PE likely” had a PE prevalence of 15.9% (20/126 encounters). This compares to 8.1% (6/74) and 15.6% (20/128) based on chart review. By chart review, the prevalence of positive Wells score components ranged from 3.5% (7/202) for hemoptysis to 44% (87/202) for tachycardia (Table 1).

**Table 1:** Proportion of Wells score components captured by the automated process.

Wells score components	Present on chart review, n/N (prevalence rate %)	Captured by automated process, n/N (sensitivity rate %)	Erroneously captured, n/N (false positive rate %)
Clinical signs or symptoms of DVT^a^ (3 points)	28/202 (14)	15/28 (54)	4/174 (2.3)
Pulse >100 (1.5 points)	87/202 (44)	87/87 (100)	1/115 (0.9)
Immobilization x 3 days or surgery in previous 4 weeks (1.5 points)	22/202 (11)	15/22 (68)	0/180 (0)
History of PE^b^ or DVT (1.5 points)	32/202 (16)	29/32 (91)	5/170 (2.9)
Hemoptysis (1 point)	7/202 (3.5)	6/7 (86)	1/195 (0.5)
Malignancy with treatment within 6 months or palliative (1 point)	47/202 (23)	41/47 (87)	8/155 (5.2)
PE as likely or more than alternate diagnoses (3 points, assumed to be true)	202/202 (100)	202/202 (100)	0/0 (0)

^a^DVT: deep venous thrombosis.

^b^PE: pulmonary embolism.

### Sensitivity of the Automated Process

Of these components, the sensitivity of the automated process ranged from 54% (15/28) for signs and symptoms of DVT, to 100% (87/87) for pulse greater than 100. Of the 13 instances where signs and symptoms of DVT were missed by the automated process (out of the 28 found by chart review), 1 was due to a description not covered by our search strategy and 12 were due to descriptions not being present in the “Chief Complaint” field of the ED nurse triage note, but only in the “History of Present Illness” or “Physical Exam” field of the ED provider note (Table S2 in Multimedia Appendix 1). Moreover, 7 out of 22 instances of immobility were only described in the “History of Present Illness” fields of ED provider notes and not captured in intensive care unit stays, operative notes, or quadriplegia diagnoses.

### Specificity of the Automated Process

False positive rates were low across all automated score components, ranging from 0% (0/180) for immobility to 5.2% (8/155) for active malignancy, corresponding to specificities of 94.8% to 100%. Several false positive findings of diagnoses of PE/DVT or active malignancy were due to erroneous entries in the problem list for the former, and inactive, past diagnoses for the latter. For all individual score components, excluding clinical gestalt, which was assumed positive in all cases, overall accuracy was 96% (1163/1212).

### Overall Performance of the Automated Process

With respect to classification of the patient as “PE likely,” the automated process achieved an accuracy of 92.1% (186 correct classifications out of 202 encounters) when compared to chart review, with sensitivity of 93% (119/128) and specificity of 90% (67/74) (Table 2 and Table 3). Positive predictive value was 94.4% (119/126), and negative predictive value was 88% (67/76). Out of a total of 202 patient encounters, there were 16 (7.9%) instances where there was discrepancy between automated and manual classifications. Moreover, 9 false negatives included 5 where signs and symptoms of DVT were present but not mentioned in the ED nurse triage note, 2 where history of PE was not documented in the problem list but was described by the ED provider note, and 2 where patients had recent surgeries described in notes but were not captured by operative notes in the EHR. In addition, 7 false positives included 3 where a search phrase was present in the ED nurse triage note but was preceded by words of negation, 3 due to erroneous entries of PE or DVT in the EHR problem list, and 1 due to an erroneous pulse entry.

**Table 2:** Performance of the automated risk classifications—confusion matrix.

Total 202 encounters	PE^a^ likely by chart review (n=128)	PE unlikely by chart review (n=74)
PE likely by automated process	119	7
PE unlikely by automated process	9	67

^a^PE: pulmonary embolism.

**Table 3:** Automated process performance measures.

Performance measure	Formula	Value
Accuracy	(119 + 67) / (119 + 7 + 9 + 67)	92.1%
Sensitivity	119 / (119 + 9)	93.0%
Specificity	67 / (67 + 7)	90.5%
Positive predictive value	119 / (119 + 7)	94.4%
Negative predictive value	67 / (67 + 9)	88.2%

### Guideline Concordance

Based on the automated process, 151 of 202 CTPA orders were guideline concordant, resulting in a concordance rate of 74.8%, compared to 153 of 202 (75.7%) based on chart review. Of the 76 cases classified as “PE unlikely” by the automated Wells score, 28 (37%) had a D-dimer ordered, with 25 (33%) resulting above the upper limit of normal. The 3 cases (1.5%) where D-dimer was within normal range and the 48 cases (24%) where no D-dimer was ordered prior to CTPA were considered nonguideline concordant.

## Discussion

### Principal Findings

In this study we created an automated process to calculate Wells score with the aim of improving CDS tool usability and evaluating provider guideline concordance. We found that our process was 96% accurate with respect to individual instances of score components, and 92% accurate with respect to risk classification when compared to a manual chart review standard. The process achieved high positive predictive value (94.4%) while preserving negative predictive value (88%). To address 2 important score components that tend to reside outside of structured EHR elements (signs and symptoms of DVT and hemoptysis), we designed an innovative key phrase search method that made use of free-text fields in notes without requiring advanced natural language processing (NLP) techniques. The automated score was able to stratify risk within ED encounters where CPTA was performed, with cases classified as “PE likely” having a CTPA yield that is double the yield of cases classified as “PE unlikely.”

Significant prior work has been carried out for the automated calculation of clinical risk prediction scores using EHR data. Much success has been achieved for scores whose variables can be exclusively derived from structured data, such as CURB-65 (confusion, uremia, respiratory rate, BP, age ≥65 years) for pneumonia severity [20], CHADS2-VASc (congestive heart failure, hypertension, age ≥75 years, diabetes mellitus, stroke, vascular disease, age 65-74 years, sex category [female]) for stroke risk [21], PESI (pulmonary embolism severity index) for PE severity [22], SOFA (sequential organ failure assessment) in sepsis [23], and the Padua prediction score for risk of venous thromboembolism [16,24]. These studies in general found very high degrees of accuracy when compared to scores derived manually, with near perfect accuracy with regards to scalar variables such as vital signs or laboratory values.

Certain isolated areas of lower sensitivity and specificity are related to the incompleteness of administrative and EHR databases of diagnoses—a well-recognized issue [24,25]—as well as the temporality of EHR-documented diagnoses, due to the accumulation of resolved, inactive medical problems and incomplete documentation of recent, acute ones. For example, Navar-Boggan et al [20] found lower specificity for active stroke risk factors in the CHA2DS2-VASc score due to detection of historical, resolved diagnoses, and Pavon et al [15] found lower sensitivity for active cancer, infection, myocardial infarction, and stroke diagnoses for the Padua score due to election of only using items from the “Admitting Diagnoses” list. The issue of temporality also affected the active malignancy component of our automatic score, which resulted in 8 false positives due to the detection of inactive, resolved cancer. Using EHR problem list diagnoses relies on their accuracy, which may introduce error [18], as it did in our score when false positive history of PE or DVT were introduced by erroneous entries, although the false positive rate was very low (2.9%).

More challenging is the extraction of information necessary for variable components more likely to be found in unstructured data, such as signs and symptoms of DVT in our study. Significant work has been carried out to address this issue, with Grouin et al [26] using NLP techniques to extract criteria for the CHA2DS2-VASc score from clinical notes, including negation and speculation handling, achieving an accuracy of 97.6% for score components and 85.7% for scores. Deleger et al [27] similarly used NLP techniques to extract data from ED physician notes and incorporated structured lab values to calculate an automated pediatric appendicitis score. The automated score achieved a sensitivity of 86.9% and positive predictive value of 86.3% when compared to manual chart review. Without the option to implement advanced NLP techniques on EHR data in real time, we devised a less complex and more transparent search strategy using a list of phrases generated from combinations of possible descriptors of signs and symptoms of DVT and hemoptysis. With the additional constraint of being only applied to EHR documentation present prior to ED CTPA ordering, our process achieved sensitivity, specificity, and overall accuracy of 54%, 98%, and 90.6% percent for signs and symptoms of DVT, and 86%, 99%, and 96.5% for hemoptysis when compared to chart review.

While our automated process shows remarkable promise for use in CDS for CTPA ordering for PE, given its high accuracy and positive and negative predictive values, it also reveals the limits of automated information retrieval. False positives and negatives are likely to occur when applied to hundreds of cases, and the automated process is naive regarding information not documented in its targeted fields. Therefore, in addition to further refining our automated process, the practical limitations of its information retrieval may be better remedied with a hybrid approach combining automated variables that are captured with high accuracy and manual input variables, as suggested by Pavon et al [15] in their study of automated Padua scores for venous thromboembolism risk classification. Additionally, inaccuracies in information retrieval need to be checked by human knowledge. After the clinician interviews and examines the patient, he or she is likely to have gathered pertinent information not captured by the EHR, information that can rectify false positives and false negatives presented by automated information retrieval. Ideally, a CDS tool incorporating the automated Wells score should also display the source of each extracted element to create transparency and enable validation by the human user.

### Limitations

Our study has several limitations. We did not study ED encounters where PE may have been considered as a diagnosis but CTPA was ultimately not performed; therefore we do not know the performance of our score in such patients. Our study was retrospective in nature, relying on manual chart review to establish a standard against which our automatic score was compared. Although our chart review was thorough, it likely represents an imperfect standard due to likely incomplete documentation of information available to the provider at the time of CTPA ordering. Our study was performed using data from 2 urban tertiary care hospitals within a single New York health care system, using Allscripts Sunrise EHR, and may not be generalizable to other settings.

### Conclusions

We successfully designed and validated an automated process to calculate a Wells criteria score for PE to risk-stratify patients prior to CTPA. Our study achieved high accuracy as well as positive and negative predictive values, demonstrating its potential for use in augmenting CDS tools with automated information retrieval. When implemented in a CDS tool, our score could serve as the foundation for a hybrid approach combining automated prepopulation of variables with the option for manual input by the provider. Our method was novel in using a keyword search strategy using a list of phrases formed from combinations of terms used to describe signs and symptoms of DVT, applied to existing EHR notes prior to CTPA ordering.

## Appendix

Multimedia Appendix 1 Supplemental material.

## Abbreviations

CDS: clinical decision support
CHADS2-VASc: congestive heart failure, hypertension, age ≥75 years, diabetes mellitus, stroke, vascular disease, age 65-74 years, sex category (female)
CTPA: computed tomography pulmonary angiography
CURB-65: confusion, uremia, respiratory rate, BP, age ≥65 years
DVT: deep venous thrombosis
ED: emergency department
EHR: electronic health record
ICD: International Classification of Disease
NLP: natural language processing
PE: pulmonary embolism
PESI: pulmonary embolism severity index
SOFA: sequential organ failure assessment
