# Supplementary material for: Automated Pulmonary Embolism Risk Assessment Using the Wells Criteria: Validation Study
Source: JMIR Form Res. 2022 Feb 28;6(2):e32230. doi: 10.2196/32230 (PMC8922138; doi:10.2196/32230)
Supplement: Multimedia Appendix 1 [file formative_v6i2e32230_app1.docx]

**Supplemental Table 1. Search Terms for Signs and Symptoms of DVT**

| LE swelling | edema to lower extremity | edema to right lower extremity | edema to L lower leg |
| --- | --- | --- | --- |
| lower extremity swelling | pain in lower extremity | pain in right lower extremity | pain in L lower leg |
| leg swelling | swollen lower extremity | swollen right lower extremity | swollen L lower leg |
| lower leg swelling | edema to leg | edema to left lower extremity | edema to R lower leg |
| calf swelling | pain in leg | pain in left lower extremity | pain in R lower leg |
| thigh swelling | swollen leg | swollen left lower extremity | swollen R lower leg |
| RLE swelling | edema to lower leg | edema to L lower extremity | edema to bilateral calf |
| LLE swelling | pain in lower leg | pain in L lower extremity | pain in bilateral calf |
| BLE swelling | swollen lower leg | swollen L lower extremity | swollen bilateral calf |
| LE swollen | edema to calf | edema to R lower extremity | edema to b/l calf |
| lower extremity swollen | pain in calf | pain in R lower extremity | pain in b/l calf |
| leg swollen | swollen calf | swollen R lower extremity | swollen b/l calf |
| lower leg swollen | edema to thigh | edema to bilateral leg | edema to bl calf |
| calf swollen | pain in thigh | pain in bilateral leg | pain in bl calf |
| thigh swollen | swollen thigh | swollen bilateral leg | swollen bl calf |
| RLE swollen | edema to RLE | edema to b/l leg | edema to right calf |
| LLE swollen | pain in RLE | pain in b/l leg | pain in right calf |
| BLE swollen | swollen RLE | swollen b/l leg | swollen right calf |
| LE edema | edema to LLE | edema to bl leg | edema to left calf |
| lower extremity edema | pain in LLE | pain in bl leg | pain in left calf |
| leg edema | swollen LLE | swollen bl leg | swollen left calf |
| lower leg edema | edema to BLE | edema to right leg | edema to L calf |
| calf edema | pain in BLE | pain in right leg | pain in L calf |
| thigh edema | swollen BLE | swollen right leg | swollen L calf |
| RLE edema | edema to bilateral LE | edema to left leg | edema to R calf |
| LLE edema | pain in bilateral LE | pain in left leg | pain in R calf |
| BLE edema | swollen bilateral LE | swollen left leg | swollen R calf |
| LE pain | edema to b/l LE | edema to L leg | edema to bilateral thigh |
| lower extremity pain | pain in b/l LE | pain in L leg | pain in bilateral thigh |
| leg pain | swollen b/l LE | swollen L leg | swollen bilateral thigh |
| lower leg pain | edema to bl LE | edema to R leg | edema to b/l thigh |
| calf pain | pain in bl LE | pain in R leg | pain in b/l thigh |
| thigh pain | swollen bl LE | swollen R leg | swollen b/l thigh |
| RLE pain | edema to right LE | edema to bilateral lower leg | edema to bl thigh |
| LLE pain | pain in right LE | pain in bilateral lower leg | pain in bl thigh |
| BLE pain | swollen right LE | swollen bilateral lower leg | swollen bl thigh |
| LE discomfort | edema to left LE | edema to b/l lower leg | edema to right thigh |
| lower extremity discomfort | pain in left LE | pain in b/l lower leg | pain in right thigh |
| leg discomfort | swollen left LE | swollen b/l lower leg | swollen right thigh |
| lower leg discomfort | edema to bilateral lower extremity | edema to bl lower leg | edema to left thigh |
| calf discomfort | pain in bilateral lower extremity | pain in bl lower leg | pain in left thigh |
| thigh discomfort | swollen bilateral lower extremity | swollen bl lower leg | swollen left thigh |
| RLE discomfort | edema to b/l lower extremity | edema to right lower leg | edema to L thigh |
| LLE discomfort | pain in b/l lower extremity | pain in right lower leg | pain in L thigh |
| BLE discomfort | swollen b/l lower extremity | swollen right lower leg | swollen L thigh |
| edema to LE | edema to bl lower extremity | edema to left lower leg | edema to R thigh |
| pain in LE | pain in bl lower extremity | pain in left lower leg | pain in R thigh |
| swollen LE | swollen bl lower extremity | swollen left lower leg | swollen R thigh |

**Supplemental Table 2. Instances of False Negatives for Automated Detection of Signs/Symptoms of DVT**

| ED Nurse Triage Note: swelling to right lower extremity |
| --- |
| ED Provider Note – HPI: “+b/l le edema R > L” |
| ED Provider Note – HPI: “L calf cramping a day ago” |
| ED Provider Note – HPI: “LLE pain” |
| ED Provider Note – HPI: “left calf/posterior knee pain” |
| ED Provider Note – HPI: “left leg pain” |
| ED Provider Note – HPI: “worsening right calf pain” |
| ED Provider Note – HPI: “LLE redness, swelling extending to L foot” |
| ED Provider Note – Physical Exam: “+ bilateral calf tenderness” |
| ED Provider Note – Physical Exam: “Mild left LE swelling” |
| ED Provider Note – Physical Exam: “mild swelling in R leg” |
| ED Provider Note – Physical Exam: “b/l pitting edema R > L” |
| ED Provider Note – Physical Exam: “LLE 2+ pitting edema greater than trace RLE pitting edema” |
